# 3β-Acet­oxy-lup-20(29)-en-28-yl 1*H*-1,2,4-tri­azole-1-carboxyl­ate

**DOI:** 10.1107/S1600536810043515

**Published:** 2010-10-31

**Authors:** R.C. Santos, A. Matos Beja, J. A. R. Salvador, J. A. Paixão

**Affiliations:** a Laboratório de Química Farmacêutica, Faculdade de Farmácia, Universidade de Coimbra, Pólo das Ciências da Saúde, Azinhaga de Santa Comba, P-3000-548 Coimbra, Portugal; b CEMDRX, Departamento de Física, Faculdade de Ciências e Tecnologia, Universidade de Coimbra, P-3004-516 Coimbra, Portugal

## Abstract

The title triterpene, C_35_H_53_N_3_O_4_, is a C-28 carbamate derivative of 3β-acet­oxy­betulin prepared in a one-step reaction from the commercially available 1,1′-carbonyl-di(1,2,4-triazole) (CDT), crystallized from acetone/*n*-hexane. All rings are *trans* fused. The carbamate and acetate substituents are in axial and equatorial positions, respectively. A quantum chemical *ab initio* Roothaan Hartree–Fock calculation of the equilibrium geometry of the isolated mol­ecule gives values for bond lengths and valency angles in close agreement with experimental values. The calculation also reproduces the observed mol­ecular conformation, with puckering parameters that agree well with those determined from the crystallographic study.

## Related literature

For the cytotoxic activity of penta­cyclic triterpenoids, see: Petronelli *et al.* (2009 ▶); Fulda (2009 ▶); Salvador (2010 ▶). For the biological activity of betulin and betulinic acid, see: Dzubak *et al.* (2006 ▶); Tolstikova *et al.* (2006 ▶). For the synthesis of carbamate derivatives of betulin and betulinic acid, see: Santos *et al.* (2009 ▶, 2010*b* ▶). For related structures, see Santos *et al.* (2010*a* ▶). For puckering and asymmetry parameters, see Cremer & Pople (1975 ▶); Duax & Norton (1975 ▶). The quantum chemical calculations were performed with the computer program *GAMESS* (Schmidt *et al.*, 1993 ▶).
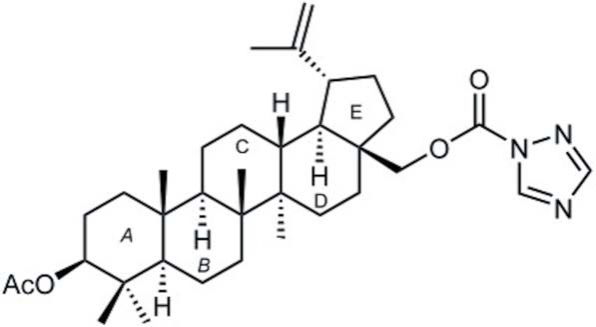


## Experimental

### 

#### Crystal data

C_35_H_53_N_3_O_4_
*M* *_r_* = 579.80
Orthorhombic, 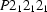

*a* = 9.2108 (4) Å
*b* = 15.5383 (6) Å
*c* = 22.9270 (9) Å
*V* = 3281.3 (2) Å^3^
*Z* = 4
Mo *K*α radiation
μ = 0.08 mm^−1^
*T* = 293 K
0.28 × 0.24 × 0.23 mm

#### Data collection

Bruker APEXII CCD area-detector diffractometer
Absorption correction: multi-scan (*SADABS*; Sheldrick, 2000 ▶) *T*_min_ = 0.880, *T*_max_ = 1.00
61378 measured reflections
4625 independent reflections
3264 reflections with *I* > 2σ(*I*)
*R*_int_ = 0.046

#### Refinement

*R*[*F* ^2^ > 2σ(*F* ^2^)] = 0.047
*wR*(*F* ^2^) = 0.137
*S* = 1.02
4625 reflections
386 parameters
H-atom parameters constrained
Δρ_max_ = 0.27 e Å^−3^
Δρ_min_ = −0.26 e Å^−3^

### 

Data collection: *APEX2* (Bruker, 2006 ▶); cell refinement: *SAINT* (Bruker, 2006 ▶); data reduction: *SAINT*; program(s) used to solve structure: *SHELXS97* (Sheldrick, 2008 ▶); program(s) used to refine structure: *SHELXL97* (Sheldrick, 2008 ▶); molecular graphics: *PLATON* (Spek, 2009 ▶); software used to prepare material for publication: *SHELXL97*.

## Supplementary Material

Crystal structure: contains datablocks global, I. DOI: 10.1107/S1600536810043515/bt5389sup1.cif

Structure factors: contains datablocks I. DOI: 10.1107/S1600536810043515/bt5389Isup2.hkl

Additional supplementary materials:  crystallographic information; 3D view; checkCIF report

## Figures and Tables

**Fig. 1.**
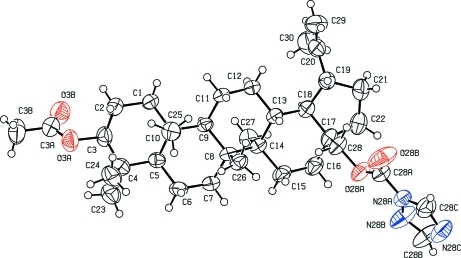
ORTEPII plot of the title compound showing the atomic numbering scheme. Displacement ellipsoids are drawn at the 50% level. H atoms are depicted as spheres of arbitrary radii.

## supplementary crystallographic information

### Comment

Pentacyclic triterpenoids are a class of pharmacologically active and structurally rich natural products with privileged motifs for further modifications and structure–activity relationship (SAR) analyses (Petronelli *et al.*, 2009). Some natural triterpenoids such as betulin and betulinic acid have shown remarkable effects in suppressing tumorigenesis as well as in inhibiting tumor (Fulda, 2009). Recently, we focused our attention on the synthesis of lupane-type carbamates and *N*-acylheterocyclic bearing derivatives. Our results showed that addition of an heterocyclic moiety at the C-3 and/or C-28 positions of betulin and betulinic acid can result in more potent *in vitro* anticancer agents than betulinic acid, with IC_50_ values between 0.8 and 28.2 µ*M*, in some human cancer cell lines of different tumor types (Santos *et al.*, 2009, 2010*b*). The general procedure for the synthesis of the novel lupane derivatives involved dissolution of the corresponding lupanes and CDI, CBMI or CDT in THF at reflux, under N_2_ (Santos *et al.*, 2009, 2010*b*). In this case the reaction of 3β-acetoxybetulin with CDT afforded the carbamate derivative 3β-acetoxy-lup-20 (29)-en-28-yl-1*H*-1,2,4-triazole-1-carboxylate in good yield (Santos *et al.*, 2010*b*).

Mindful of the biological and synthetic importance of such molecules, we report in this communication the molecular structure of the 3β-acetoxy-lup-20 (29)-en-28-yl-1*H*-1,2,4-triazole-1-carboxylate determined by single-crystal X-ray diffraction, and compare it with that of the free molecule as given by quantum mechanical *ab initio* calculation. The structure of this compound with the corresponding atomic numbering scheme is shown in Fig. 1.

All six-membered rings are fused *trans* and have slightly distorted chair conformations; the 5-membered ring adopts a twisted conformation around C17—C18, as shown by the Cremer & Pople (1975) parameters: [ring A: Q = 0.565 (3) Å, θ = 3.4 (3)° and φ = 129 (5)°; B: Q = 0.569 (3) Å, θ = 8.9 (3)° and φ = 17.9 (18)°; C: Q = 0.605 (3) Å, θ = 7.9 (3)° and φ = 323.7 (19)°; D: Q = 0.558 (3) Å, θ = 173.7 (3)° and φ = 80 (3)°; E: q_2_ = 0.451 (3) Å, φ_2_ = 9.6 (4)°].

The carbamate and acetate substituints are in axial and equatorial positions, respectively.

In order to gain some insight on how the crystal packing of (I) might affect the molecular geometry we have performed a quantum chemical calculation on the equilibrium geometry of the free molecule. These *ab initio* calculations reproduce well the observed bond length and valency angles of the molecule with the exception of bonds C30—C20 [obs: 1.467 (5) calc: 1.511 Å] and N28—C28B [obs: 1.331 (6) calc: 1.366 Å] Also, the calculated conformation of the rings are very close to the experimental values. The conformation of the molecule mainly differs from a small rotation of the triazole-carboxylate substituent around the C28–O28A bond as shown by the values of the C28—O28A—C28A—O28B torsion angle [obs: 149.6 (3) calc: 179.4]

There are no strong hydrogen bonds in the crystal structure, due to the lack of strong H-donors.

### Experimental

The synthesis of 3β-acetoxy-lup-20 (29)-en-28-yl-1*H*-1,2,4-triazole-1-carboxylate was efficiently accomplished by reaction with CDT (Santos *et al.*, 2009). The product of this reaction was isolated in 74% yield and identified as the title compound from MS, IR, 1H and 13C NMR spectroscopy data (Santos *et al.*, 2010*b*). Recrystallization from acetone/n-hexane at room temperature gave colourless single crystals suitable for X-ray diffraction analysis.

The *ab initio* calculations were performed with the computer program GAMESS (Schmidt *et al.*, 1993). A molecular orbital Roothaan Hartree-Fock method was used with an extended 6-31 G(d,p) basis set. Tight conditions for convergence of both the self-consistent field cycles and maximum density and energy gradient variations were imposed (10^-6^ atomic units). The program was run on the Milipeia cluster of UC-LCA (using 16 Opteron cores, 2.2 GHz running Linux).

### Refinement

All H atoms were refined as riding on their parent atoms using *SHELXL97* defaults. The absolute configuration was not determined from the X-ray data, as the molecule lacks any strong anomalous scatterer atom at the Mo Kα wavelength, but was known from the synthetic route. Friedel pairs of reflections (2247 pairs) were merged before refinement.

### Crystal data

**Table:**

C_35_H_53_N_3_O_4_	*D*_x_ = 1.174 Mg m^−^^3^
*M**_r_* = 579.80	Melting point: 386 K
Orthorhombic, *P*2_1_2_1_2_1_	Mo *K*α radiation, λ = 0.71073 Å
*a* = 9.2108 (4) Å	Cell parameters from 5792 reflections
*b* = 15.5383 (6) Å	θ = 2.6–22.2°
*c* = 22.9270 (9) Å	µ = 0.08 mm^−^^1^
*V* = 3281.3 (2) Å^3^	*T* = 293 K
*Z* = 4	Triangular prism, colourless
*F*(000) = 1264	0.28 × 0.24 × 0.23 mm

### Data collection

**Table:**

Bruker APEXII CCD area-detector diffractometer	4625 independent reflections
Radiation source: fine-focus sealed tube	3264 reflections with *I* > 2σ(*I*)
graphite	*R*_int_ = 0.046
φ and ω scans	θ_max_ = 28.5°, θ_min_ = 1.6°
Absorption correction: multi-scan (*SADABS*; Sheldrick, 2000)	*h* = −12→12
*T*_min_ = 0.880, *T*_max_ = 1.00	*k* = −20→20
61378 measured reflections	*l* = −30→30

### Refinement

**Table:**

Refinement on *F*^2^	Primary atom site location: structure-invariant direct methods
Least-squares matrix: full	Secondary atom site location: difference Fourier map
*R*[*F*^2^ > 2σ(*F*^2^)] = 0.047	Hydrogen site location: inferred from neighbouring sites
*wR*(*F*^2^) = 0.137	H-atom parameters constrained
*S* = 1.01	*w* = 1/[σ^2^(*F*_o_^2^) + (0.0682*P*)^2^ + 0.4749*P*] where *P* = (*F*_o_^2^ + 2*F*_c_^2^)/3
4625 reflections	(Δ/σ)_max_ < 0.001
386 parameters	Δρ_max_ = 0.27 e Å^−^^3^
0 restraints	Δρ_min_ = −0.26 e Å^−^^3^

### Special details

**Table:**

Geometry. All e.s.d.'s (except the e.s.d. in the dihedral angle between two l.s. planes) are estimated using the full covariance matrix. The cell e.s.d.'s are taken into account individually in the estimation of e.s.d.'s in distances, angles and torsion angles; correlations between e.s.d.'s in cell parameters are only used when they are defined by crystal symmetry. An approximate (isotropic) treatment of cell e.s.d.'s is used for estimating e.s.d.'s involving l.s. planes.
Refinement. Refinement of *F*^2^ against ALL reflections. The weighted *R*-factor *wR* and goodness of fit *S* are based on *F*^2^, conventional *R*-factors *R* are based on *F*, with *F* set to zero for negative *F*^2^. The threshold expression of *F*^2^ > σ(*F*^2^) is used only for calculating *R*-factors(gt) *etc*. and is not relevant to the choice of reflections for refinement. *R*-factors based on *F*^2^ are statistically about twice as large as those based on *F*, and *R*-factors based on ALL data will be even larger.

### Fractional atomic coordinates and isotropic or equivalent isotropic displacement parameters (Å^2^)

**Table:**

	*x*	*y*	*z*	*U*_iso_*/*U*_eq_	
C1	0.6151 (3)	−0.06751 (16)	0.10936 (12)	0.0579 (7)	
H1A	0.7006	−0.1040	0.1092	0.069*	
H1B	0.5885	−0.0574	0.1497	0.069*	
C2	0.4912 (4)	−0.11521 (19)	0.07917 (14)	0.0684 (8)	
H2A	0.5209	−0.1317	0.0402	0.082*	
H2B	0.4683	−0.1672	0.1007	0.082*	
C3	0.3584 (3)	−0.05852 (19)	0.07579 (12)	0.0614 (7)	
H3	0.3280	−0.0444	0.1156	0.074*	
C4	0.3822 (3)	0.02562 (18)	0.04263 (11)	0.0554 (6)	
C5	0.5098 (3)	0.07087 (16)	0.07423 (10)	0.0471 (5)	
H5	0.4753	0.0779	0.1144	0.057*	
C6	0.5400 (3)	0.16314 (17)	0.05369 (12)	0.0563 (7)	
H6A	0.5908	0.1617	0.0166	0.068*	
H6B	0.4488	0.1932	0.0480	0.068*	
C7	0.6316 (3)	0.21111 (16)	0.09849 (12)	0.0556 (6)	
H7A	0.5769	0.2156	0.1345	0.067*	
H7B	0.6490	0.2691	0.0844	0.067*	
C8	0.7783 (3)	0.16820 (14)	0.11152 (10)	0.0446 (5)	
C26	0.8744 (3)	0.18187 (17)	0.05709 (10)	0.0548 (6)	
H26A	0.8204	0.1671	0.0227	0.082*	
H26B	0.9589	0.1459	0.0598	0.082*	
H26C	0.9036	0.2411	0.0550	0.082*	
C9	0.7526 (3)	0.07045 (14)	0.12451 (9)	0.0433 (5)	
H9	0.6988	0.0697	0.1614	0.052*	
C10	0.6540 (3)	0.01952 (15)	0.08079 (10)	0.0465 (5)	
C25	0.7321 (3)	0.00105 (19)	0.02266 (11)	0.0613 (7)	
H25A	0.6960	−0.0517	0.0064	0.092*	
H25B	0.8346	−0.0040	0.0295	0.092*	
H25C	0.7144	0.0474	−0.0041	0.092*	
C11	0.8960 (3)	0.02553 (15)	0.13842 (11)	0.0502 (6)	
H11A	0.8764	−0.0338	0.1488	0.060*	
H11B	0.9562	0.0253	0.1037	0.060*	
C12	0.9794 (3)	0.06810 (16)	0.18781 (11)	0.0531 (6)	
H12A	1.0733	0.0405	0.1918	0.064*	
H12B	0.9267	0.0600	0.2240	0.064*	
C13	1.0015 (3)	0.16425 (15)	0.17724 (10)	0.0466 (5)	
H13	1.0561	0.1695	0.1407	0.056*	
C14	0.8528 (3)	0.21018 (15)	0.16712 (10)	0.0472 (5)	
C27	0.7567 (3)	0.20149 (19)	0.22234 (12)	0.0619 (7)	
H27A	0.8041	0.2288	0.2547	0.093*	
H27B	0.7419	0.1417	0.2310	0.093*	
H27C	0.6646	0.2286	0.2155	0.093*	
C15	0.8773 (4)	0.30855 (15)	0.15862 (13)	0.0604 (7)	
H15A	0.9208	0.3178	0.1206	0.072*	
H15B	0.7835	0.3369	0.1586	0.072*	
C16	0.9732 (4)	0.35123 (18)	0.20470 (13)	0.0652 (8)	
H16A	0.9896	0.4109	0.1941	0.078*	
H16B	0.9238	0.3502	0.2420	0.078*	
C17	1.1182 (3)	0.30553 (16)	0.21027 (11)	0.0559 (6)	
C18	1.0915 (3)	0.20927 (16)	0.22413 (11)	0.0512 (6)	
H18	1.0331	0.2079	0.2598	0.061*	
C19	1.2424 (3)	0.17477 (17)	0.24085 (11)	0.0565 (6)	
H19	1.2955	0.1624	0.2048	0.068*	
C21	1.3161 (4)	0.2544 (2)	0.27089 (16)	0.0756 (9)	
H21A	1.3307	0.2435	0.3121	0.091*	
H21B	1.4095	0.2663	0.2531	0.091*	
C22	1.2130 (4)	0.3306 (2)	0.26217 (14)	0.0734 (9)	
H22A	1.1542	0.3398	0.2967	0.088*	
H22B	1.2670	0.3828	0.2539	0.088*	
O28B	1.4367 (4)	0.38506 (16)	0.09786 (15)	0.1169 (11)	
O28A	1.2328 (2)	0.40742 (11)	0.14684 (10)	0.0707 (6)	
C28	1.2065 (4)	0.31540 (16)	0.15439 (13)	0.0631 (8)	
H28A	1.2978	0.2846	0.1576	0.076*	
H28B	1.1531	0.2926	0.1214	0.076*	
C28A	1.3511 (3)	0.43009 (17)	0.12090 (12)	0.0579 (7)	
C30	1.1593 (5)	0.0930 (3)	0.33192 (15)	0.1015 (13)	
H30A	1.0586	0.0914	0.3211	0.152*	
H30B	1.1776	0.1434	0.3550	0.152*	
H30C	1.1827	0.0426	0.3542	0.152*	
C29	1.3388 (5)	0.0314 (2)	0.26551 (18)	0.0934 (12)	
H29A	1.3455	−0.0164	0.2897	0.112*	
H29B	1.3947	0.0345	0.2318	0.112*	
C20	1.2492 (4)	0.0955 (2)	0.27912 (12)	0.0679 (8)	
N28A	1.3661 (3)	0.52003 (13)	0.11931 (9)	0.0551 (5)	
C28C	1.4730 (4)	0.5648 (2)	0.09318 (16)	0.0800 (10)	
H28C	1.5497	0.5400	0.0729	0.096*	
N28C	1.4553 (4)	0.64662 (19)	0.09998 (13)	0.0913 (10)	
C28B	1.3339 (6)	0.6496 (2)	0.13141 (16)	0.1047 (16)	
H28D	1.2941	0.7015	0.1437	0.126*	
N28B	1.2732 (4)	0.57561 (17)	0.14415 (13)	0.0927 (10)	
O3A	0.2407 (3)	−0.10679 (15)	0.04757 (9)	0.0766 (6)	
C3A	0.1539 (4)	−0.1536 (2)	0.08194 (17)	0.0746 (9)	
C3B	0.0438 (5)	−0.2023 (3)	0.0477 (2)	0.1178 (17)	
H3B1	−0.0081	−0.2405	0.0731	0.177*	
H3B2	0.0916	−0.2350	0.0178	0.177*	
H3B3	−0.0231	−0.1627	0.0300	0.177*	
O3B	0.1642 (3)	−0.15572 (16)	0.13343 (11)	0.0893 (7)	
C23	0.2444 (4)	0.0802 (2)	0.04879 (15)	0.0751 (9)	
H23A	0.1617	0.0470	0.0367	0.113*	
H23B	0.2525	0.1305	0.0247	0.113*	
H23C	0.2326	0.0972	0.0888	0.113*	
C24	0.4052 (4)	0.0100 (2)	−0.02329 (11)	0.0722 (9)	
H24A	0.4741	−0.0357	−0.0287	0.108*	
H24B	0.4413	0.0616	−0.0411	0.108*	
H24C	0.3145	−0.0057	−0.0410	0.108*	

### Atomic displacement parameters (Å^2^)

**Table:**

	*U*^11^	*U*^22^	*U*^33^	*U*^12^	*U*^13^	*U*^23^
C1	0.0710 (18)	0.0417 (12)	0.0609 (14)	−0.0054 (13)	−0.0163 (14)	0.0070 (11)
C2	0.081 (2)	0.0515 (15)	0.0729 (18)	−0.0131 (16)	−0.0204 (17)	0.0042 (13)
C3	0.0663 (18)	0.0673 (17)	0.0506 (13)	−0.0177 (15)	−0.0091 (13)	−0.0030 (12)
C4	0.0584 (16)	0.0628 (16)	0.0450 (12)	0.0015 (14)	−0.0051 (12)	0.0002 (11)
C5	0.0529 (14)	0.0493 (13)	0.0391 (11)	0.0029 (12)	−0.0007 (11)	0.0023 (10)
C6	0.0595 (17)	0.0511 (15)	0.0585 (14)	0.0087 (13)	−0.0062 (13)	0.0123 (12)
C7	0.0610 (16)	0.0405 (12)	0.0653 (15)	0.0070 (13)	−0.0060 (14)	0.0056 (11)
C8	0.0538 (15)	0.0354 (11)	0.0446 (11)	0.0047 (10)	0.0025 (11)	0.0067 (9)
C26	0.0673 (17)	0.0485 (13)	0.0486 (12)	−0.0040 (13)	0.0025 (13)	0.0091 (11)
C9	0.0534 (14)	0.0369 (10)	0.0395 (10)	0.0001 (11)	0.0002 (10)	0.0067 (8)
C10	0.0555 (14)	0.0405 (11)	0.0435 (11)	0.0016 (11)	−0.0033 (11)	0.0037 (10)
C25	0.0696 (19)	0.0622 (16)	0.0521 (13)	0.0051 (15)	0.0017 (13)	−0.0098 (12)
C11	0.0582 (15)	0.0324 (11)	0.0599 (13)	0.0004 (11)	−0.0062 (13)	0.0073 (10)
C12	0.0591 (16)	0.0415 (12)	0.0588 (14)	−0.0021 (12)	−0.0110 (12)	0.0105 (11)
C13	0.0557 (15)	0.0379 (11)	0.0460 (12)	−0.0008 (11)	0.0006 (11)	0.0022 (9)
C14	0.0564 (15)	0.0370 (11)	0.0483 (12)	0.0012 (11)	0.0037 (12)	−0.0003 (9)
C27	0.0609 (17)	0.0701 (18)	0.0546 (14)	0.0003 (15)	0.0088 (14)	−0.0073 (13)
C15	0.0696 (19)	0.0367 (12)	0.0749 (17)	0.0053 (13)	0.0024 (15)	−0.0028 (11)
C16	0.076 (2)	0.0424 (13)	0.0772 (18)	0.0000 (15)	0.0019 (16)	−0.0104 (13)
C17	0.0673 (17)	0.0403 (12)	0.0601 (14)	−0.0046 (13)	0.0043 (14)	−0.0102 (11)
C18	0.0617 (16)	0.0449 (13)	0.0472 (12)	−0.0054 (12)	0.0026 (12)	−0.0011 (10)
C19	0.0613 (16)	0.0541 (14)	0.0542 (13)	−0.0044 (13)	−0.0046 (13)	−0.0018 (11)
C21	0.075 (2)	0.0687 (19)	0.083 (2)	−0.0126 (17)	−0.0129 (18)	−0.0126 (16)
C22	0.084 (2)	0.0630 (17)	0.0735 (18)	−0.0137 (17)	−0.0030 (17)	−0.0216 (15)
O28B	0.115 (2)	0.0569 (14)	0.178 (3)	0.0232 (15)	0.069 (2)	0.0178 (16)
O28A	0.0707 (14)	0.0390 (9)	0.1026 (15)	−0.0049 (9)	0.0170 (12)	0.0013 (9)
C28	0.077 (2)	0.0369 (12)	0.0758 (18)	−0.0084 (13)	0.0056 (15)	−0.0017 (12)
C28A	0.0659 (18)	0.0434 (13)	0.0644 (15)	0.0007 (14)	0.0034 (14)	0.0015 (12)
C30	0.109 (3)	0.129 (3)	0.0663 (19)	−0.016 (3)	−0.009 (2)	0.029 (2)
C29	0.114 (3)	0.071 (2)	0.095 (3)	0.005 (2)	−0.026 (2)	0.0128 (19)
C20	0.078 (2)	0.0687 (18)	0.0573 (15)	−0.0102 (17)	−0.0224 (15)	0.0064 (13)
N28A	0.0686 (15)	0.0433 (11)	0.0534 (11)	−0.0037 (11)	0.0010 (11)	0.0030 (9)
C28C	0.077 (2)	0.069 (2)	0.095 (2)	−0.0130 (19)	0.0108 (19)	0.0151 (17)
N28C	0.129 (3)	0.0584 (16)	0.0869 (19)	−0.0260 (19)	0.008 (2)	0.0077 (14)
C28B	0.184 (5)	0.0436 (16)	0.087 (2)	−0.004 (2)	0.043 (3)	−0.0026 (15)
N28B	0.132 (3)	0.0465 (13)	0.099 (2)	0.0014 (17)	0.050 (2)	−0.0007 (14)
O3A	0.0812 (15)	0.0842 (15)	0.0645 (12)	−0.0263 (13)	−0.0180 (12)	0.0007 (11)
C3A	0.074 (2)	0.0611 (18)	0.088 (2)	−0.0109 (17)	−0.0110 (19)	0.0049 (17)
C3B	0.117 (4)	0.106 (3)	0.130 (3)	−0.053 (3)	−0.046 (3)	0.017 (3)
O3B	0.0966 (18)	0.0872 (16)	0.0840 (16)	−0.0238 (15)	−0.0001 (14)	0.0063 (13)
C23	0.0578 (18)	0.084 (2)	0.083 (2)	0.0057 (18)	−0.0044 (17)	−0.0024 (17)
C24	0.078 (2)	0.093 (2)	0.0453 (13)	−0.0016 (19)	−0.0126 (14)	−0.0007 (14)

### Geometric parameters (Å, °)

**Table:**

C1—C2	1.527 (4)	C15—H15A	0.9700
C1—C10	1.545 (3)	C15—H15B	0.9700
C1—H1A	0.9700	C16—C17	1.518 (4)
C1—H1B	0.9700	C16—H16A	0.9700
C2—C3	1.510 (4)	C16—H16B	0.9700
C2—H2A	0.9700	C17—C28	1.525 (4)
C2—H2B	0.9700	C17—C22	1.526 (4)
C3—O3A	1.468 (3)	C17—C18	1.549 (3)
C3—C4	1.528 (4)	C18—C19	1.538 (4)
C3—H3	0.9800	C18—H18	0.9800
C4—C23	1.533 (4)	C19—C20	1.514 (4)
C4—C24	1.545 (4)	C19—C21	1.570 (4)
C4—C5	1.550 (4)	C19—H19	0.9800
C5—C6	1.534 (4)	C21—C22	1.530 (5)
C5—C10	1.557 (3)	C21—H21A	0.9700
C5—H5	0.9800	C21—H21B	0.9700
C6—C7	1.524 (4)	C22—H22A	0.9700
C6—H6A	0.9700	C22—H22B	0.9700
C6—H6B	0.9700	O28B—C28A	1.179 (4)
C7—C8	1.536 (4)	O28A—C28A	1.290 (3)
C7—H7A	0.9700	O28A—C28	1.461 (3)
C7—H7B	0.9700	C28—H28A	0.9700
C8—C26	1.545 (3)	C28—H28B	0.9700
C8—C9	1.566 (3)	C28A—N28A	1.405 (3)
C8—C14	1.588 (3)	C30—C20	1.467 (5)
C26—H26A	0.9600	C30—H30A	0.9600
C26—H26B	0.9600	C30—H30B	0.9600
C26—H26C	0.9600	C30—H30C	0.9600
C9—C11	1.528 (3)	C29—C20	1.330 (5)
C9—C10	1.567 (3)	C29—H29A	0.9300
C9—H9	0.9800	C29—H29B	0.9300
C10—C25	1.541 (4)	N28A—N28B	1.343 (3)
C25—H25A	0.9600	N28A—C28C	1.346 (4)
C25—H25B	0.9600	C28C—N28C	1.292 (5)
C25—H25C	0.9600	C28C—H28C	0.9300
C11—C12	1.520 (3)	N28C—C28B	1.331 (6)
C11—H11A	0.9700	C28B—N28B	1.311 (4)
C11—H11B	0.9700	C28B—H28D	0.9300
C12—C13	1.527 (3)	O3A—C3A	1.337 (4)
C12—H12A	0.9700	C3A—O3B	1.185 (4)
C12—H12B	0.9700	C3A—C3B	1.489 (5)
C13—C18	1.527 (3)	C3B—H3B1	0.9600
C13—C14	1.562 (4)	C3B—H3B2	0.9600
C13—H13	0.9800	C3B—H3B3	0.9600
C14—C27	1.551 (4)	C23—H23A	0.9600
C14—C15	1.557 (3)	C23—H23B	0.9600
C27—H27A	0.9600	C23—H23C	0.9600
C27—H27B	0.9600	C24—H24A	0.9600
C27—H27C	0.9600	C24—H24B	0.9600
C15—C16	1.529 (4)	C24—H24C	0.9600
			
C2—C1—C10	114.0 (2)	C16—C15—C14	115.0 (2)
C2—C1—H1A	108.8	C16—C15—H15A	108.5
C10—C1—H1A	108.8	C14—C15—H15A	108.5
C2—C1—H1B	108.8	C16—C15—H15B	108.5
C10—C1—H1B	108.8	C14—C15—H15B	108.5
H1A—C1—H1B	107.7	H15A—C15—H15B	107.5
C3—C2—C1	110.2 (2)	C17—C16—C15	111.3 (2)
C3—C2—H2A	109.6	C17—C16—H16A	109.4
C1—C2—H2A	109.6	C15—C16—H16A	109.4
C3—C2—H2B	109.6	C17—C16—H16B	109.4
C1—C2—H2B	109.6	C15—C16—H16B	109.4
H2A—C2—H2B	108.1	H16A—C16—H16B	108.0
O3A—C3—C2	108.8 (2)	C16—C17—C28	110.6 (2)
O3A—C3—C4	108.9 (2)	C16—C17—C22	116.7 (2)
C2—C3—C4	114.1 (2)	C28—C17—C22	108.9 (2)
O3A—C3—H3	108.3	C16—C17—C18	109.2 (2)
C2—C3—H3	108.3	C28—C17—C18	110.7 (2)
C4—C3—H3	108.3	C22—C17—C18	100.2 (2)
C3—C4—C23	108.0 (2)	C13—C18—C19	120.4 (2)
C3—C4—C24	111.8 (2)	C13—C18—C17	112.6 (2)
C23—C4—C24	106.9 (2)	C19—C18—C17	104.1 (2)
C3—C4—C5	105.3 (2)	C13—C18—H18	106.3
C23—C4—C5	109.5 (2)	C19—C18—H18	106.3
C24—C4—C5	115.1 (2)	C17—C18—H18	106.3
C6—C5—C4	114.7 (2)	C20—C19—C18	117.7 (3)
C6—C5—C10	110.7 (2)	C20—C19—C21	111.7 (2)
C4—C5—C10	117.4 (2)	C18—C19—C21	103.0 (2)
C6—C5—H5	104.1	C20—C19—H19	108.0
C4—C5—H5	104.1	C18—C19—H19	108.0
C10—C5—H5	104.1	C21—C19—H19	108.0
C7—C6—C5	110.5 (2)	C22—C21—C19	106.5 (2)
C7—C6—H6A	109.5	C22—C21—H21A	110.4
C5—C6—H6A	109.5	C19—C21—H21A	110.4
C7—C6—H6B	109.5	C22—C21—H21B	110.4
C5—C6—H6B	109.5	C19—C21—H21B	110.4
H6A—C6—H6B	108.1	H21A—C21—H21B	108.6
C6—C7—C8	113.9 (2)	C17—C22—C21	105.0 (2)
C6—C7—H7A	108.8	C17—C22—H22A	110.7
C8—C7—H7A	108.8	C21—C22—H22A	110.7
C6—C7—H7B	108.8	C17—C22—H22B	110.7
C8—C7—H7B	108.8	C21—C22—H22B	110.7
H7A—C7—H7B	107.7	H22A—C22—H22B	108.8
C7—C8—C26	106.7 (2)	C28A—O28A—C28	117.5 (2)
C7—C8—C9	109.0 (2)	O28A—C28—C17	106.6 (2)
C26—C8—C9	111.94 (19)	O28A—C28—H28A	110.4
C7—C8—C14	110.99 (19)	C17—C28—H28A	110.4
C26—C8—C14	110.1 (2)	O28A—C28—H28B	110.4
C9—C8—C14	108.12 (17)	C17—C28—H28B	110.4
C8—C26—H26A	109.5	H28A—C28—H28B	108.6
C8—C26—H26B	109.5	O28B—C28A—O28A	127.5 (3)
H26A—C26—H26B	109.5	O28B—C28A—N28A	120.8 (3)
C8—C26—H26C	109.5	O28A—C28A—N28A	111.5 (3)
H26A—C26—H26C	109.5	C20—C30—H30A	109.5
H26B—C26—H26C	109.5	C20—C30—H30B	109.5
C11—C9—C8	110.6 (2)	H30A—C30—H30B	109.5
C11—C9—C10	113.82 (19)	C20—C30—H30C	109.5
C8—C9—C10	117.10 (18)	H30A—C30—H30C	109.5
C11—C9—H9	104.6	H30B—C30—H30C	109.5
C8—C9—H9	104.6	C20—C29—H29A	120.0
C10—C9—H9	104.6	C20—C29—H29B	120.0
C25—C10—C1	108.2 (2)	H29A—C29—H29B	120.0
C25—C10—C5	114.2 (2)	C29—C20—C30	121.6 (3)
C1—C10—C5	107.0 (2)	C29—C20—C19	119.9 (3)
C25—C10—C9	112.1 (2)	C30—C20—C19	118.4 (3)
C1—C10—C9	107.77 (18)	N28B—N28A—C28C	108.8 (3)
C5—C10—C9	107.30 (19)	N28B—N28A—C28A	124.5 (3)
C10—C25—H25A	109.5	C28C—N28A—C28A	126.7 (3)
C10—C25—H25B	109.5	N28C—C28C—N28A	111.3 (3)
H25A—C25—H25B	109.5	N28A—C28C—C28B	71.5 (2)
C10—C25—H25C	109.5	N28C—C28C—H28C	124.4
H25A—C25—H25C	109.5	N28A—C28C—H28C	124.4
H25B—C25—H25C	109.5	C28B—C28C—H28C	164.2
C12—C11—C9	113.2 (2)	C28C—N28C—C28B	101.8 (3)
C12—C11—H11A	108.9	N28B—C28B—N28C	116.7 (3)
C9—C11—H11A	108.9	N28B—C28B—C28C	78.3 (2)
C12—C11—H11B	108.9	N28B—C28B—H28D	121.7
C9—C11—H11B	108.9	N28C—C28B—H28D	121.7
H11A—C11—H11B	107.8	C28C—C28B—H28D	160.0
C11—C12—C13	112.0 (2)	C28B—N28B—N28A	101.4 (3)
C11—C12—H12A	109.2	C3A—O3A—C3	117.3 (2)
C13—C12—H12A	109.2	O3B—C3A—O3A	123.7 (3)
C11—C12—H12B	109.2	O3B—C3A—C3B	124.5 (4)
C13—C12—H12B	109.2	O3A—C3A—C3B	111.9 (3)
H12A—C12—H12B	107.9	C3A—C3B—H3B1	109.5
C18—C13—C12	114.1 (2)	C3A—C3B—H3B2	109.5
C18—C13—C14	111.80 (19)	H3B1—C3B—H3B2	109.5
C12—C13—C14	110.7 (2)	C3A—C3B—H3B3	109.5
C18—C13—H13	106.6	H3B1—C3B—H3B3	109.5
C12—C13—H13	106.6	H3B2—C3B—H3B3	109.5
C14—C13—H13	106.6	C4—C23—H23A	109.5
C27—C14—C15	105.7 (2)	C4—C23—H23B	109.5
C27—C14—C13	109.8 (2)	H23A—C23—H23B	109.5
C15—C14—C13	109.9 (2)	C4—C23—H23C	109.5
C27—C14—C8	111.9 (2)	H23A—C23—H23C	109.5
C15—C14—C8	111.42 (19)	H23B—C23—H23C	109.5
C13—C14—C8	108.10 (18)	C4—C24—H24A	109.5
C14—C27—H27A	109.5	C4—C24—H24B	109.5
C14—C27—H27B	109.5	H24A—C24—H24B	109.5
H27A—C27—H27B	109.5	C4—C24—H24C	109.5
C14—C27—H27C	109.5	H24A—C24—H24C	109.5
H27A—C27—H27C	109.5	H24B—C24—H24C	109.5
H27B—C27—H27C	109.5		
			
C10—C1—C2—C3	−55.5 (3)	C26—C8—C14—C13	−61.4 (2)
C1—C2—C3—O3A	−179.0 (2)	C9—C8—C14—C13	61.2 (2)
C1—C2—C3—C4	59.2 (3)	C27—C14—C15—C16	69.1 (3)
O3A—C3—C4—C23	64.5 (3)	C13—C14—C15—C16	−49.3 (3)
C2—C3—C4—C23	−173.7 (2)	C8—C14—C15—C16	−169.1 (2)
O3A—C3—C4—C24	−52.9 (3)	C14—C15—C16—C17	54.0 (3)
C2—C3—C4—C24	68.9 (3)	C15—C16—C17—C28	65.9 (3)
O3A—C3—C4—C5	−178.6 (2)	C15—C16—C17—C22	−168.9 (2)
C2—C3—C4—C5	−56.8 (3)	C15—C16—C17—C18	−56.2 (3)
C3—C4—C5—C6	−171.9 (2)	C12—C13—C18—C19	53.6 (3)
C23—C4—C5—C6	−56.1 (3)	C14—C13—C18—C19	−179.8 (2)
C24—C4—C5—C6	64.4 (3)	C12—C13—C18—C17	177.0 (2)
C3—C4—C5—C10	55.5 (3)	C14—C13—C18—C17	−56.3 (3)
C23—C4—C5—C10	171.3 (2)	C16—C17—C18—C13	59.0 (3)
C24—C4—C5—C10	−68.2 (3)	C28—C17—C18—C13	−63.0 (3)
C4—C5—C6—C7	162.3 (2)	C22—C17—C18—C13	−177.9 (2)
C10—C5—C6—C7	−62.0 (3)	C16—C17—C18—C19	−168.9 (2)
C5—C6—C7—C8	58.4 (3)	C28—C17—C18—C19	69.1 (3)
C6—C7—C8—C26	71.6 (3)	C22—C17—C18—C19	−45.8 (3)
C6—C7—C8—C9	−49.5 (3)	C13—C18—C19—C20	−76.5 (3)
C6—C7—C8—C14	−168.4 (2)	C17—C18—C19—C20	156.1 (2)
C7—C8—C9—C11	−179.3 (2)	C13—C18—C19—C21	160.1 (2)
C26—C8—C9—C11	62.9 (3)	C17—C18—C19—C21	32.7 (3)
C14—C8—C9—C11	−58.6 (2)	C20—C19—C21—C22	−134.6 (3)
C7—C8—C9—C10	48.0 (3)	C18—C19—C21—C22	−7.3 (3)
C26—C8—C9—C10	−69.8 (3)	C16—C17—C22—C21	158.4 (3)
C14—C8—C9—C10	168.74 (19)	C28—C17—C22—C21	−75.6 (3)
C2—C1—C10—C25	−72.6 (3)	C18—C17—C22—C21	40.7 (3)
C2—C1—C10—C5	50.9 (3)	C19—C21—C22—C17	−21.1 (3)
C2—C1—C10—C9	166.0 (2)	C28A—O28A—C28—C17	149.6 (3)
C6—C5—C10—C25	−67.9 (3)	C16—C17—C28—O28A	61.9 (3)
C4—C5—C10—C25	66.5 (3)	C22—C17—C28—O28A	−67.6 (3)
C6—C5—C10—C1	172.46 (19)	C18—C17—C28—O28A	−176.9 (2)
C4—C5—C10—C1	−53.2 (3)	C28—O28A—C28A—O28B	6.8 (5)
C6—C5—C10—C9	57.0 (2)	C28—O28A—C28A—N28A	−176.9 (2)
C4—C5—C10—C9	−168.60 (19)	C18—C19—C20—C29	133.8 (3)
C11—C9—C10—C25	−57.1 (3)	C21—C19—C20—C29	−107.4 (3)
C8—C9—C10—C25	74.1 (3)	C18—C19—C20—C30	−48.4 (4)
C11—C9—C10—C1	61.8 (3)	C21—C19—C20—C30	70.5 (4)
C8—C9—C10—C1	−167.0 (2)	O28B—C28A—N28A—N28B	179.7 (3)
C11—C9—C10—C5	176.71 (18)	O28A—C28A—N28A—N28B	3.1 (4)
C8—C9—C10—C5	−52.1 (3)	O28B—C28A—N28A—C28C	−0.1 (5)
C8—C9—C11—C12	54.8 (3)	O28A—C28A—N28A—C28C	−176.6 (3)
C10—C9—C11—C12	−170.86 (19)	N28B—N28A—C28C—N28C	0.4 (4)
C9—C11—C12—C13	−53.1 (3)	C28A—N28A—C28C—N28C	−179.8 (3)
C11—C12—C13—C18	−177.0 (2)	N28B—N28A—C28C—C28B	0.6 (3)
C11—C12—C13—C14	55.8 (3)	C28A—N28A—C28C—C28B	−179.6 (3)
C18—C13—C14—C27	−66.3 (3)	N28A—C28C—N28C—C28B	0.2 (4)
C12—C13—C14—C27	62.2 (3)	C28C—N28C—C28B—N28B	−0.9 (5)
C18—C13—C14—C15	49.6 (3)	N28C—C28C—C28B—N28B	179.2 (5)
C12—C13—C14—C15	178.0 (2)	N28A—C28C—C28B—N28B	−0.6 (3)
C18—C13—C14—C8	171.36 (18)	N28A—C28C—C28B—N28C	−179.8 (4)
C12—C13—C14—C8	−60.2 (2)	N28C—C28B—N28B—N28A	1.1 (5)
C7—C8—C14—C27	59.5 (3)	C28C—C28B—N28B—N28A	0.6 (3)
C26—C8—C14—C27	177.5 (2)	C28C—N28A—N28B—C28B	−0.9 (4)
C9—C8—C14—C27	−59.9 (3)	C28A—N28A—N28B—C28B	179.3 (3)
C7—C8—C14—C15	−58.5 (3)	C2—C3—O3A—C3A	89.2 (3)
C26—C8—C14—C15	59.4 (3)	C4—C3—O3A—C3A	−145.8 (3)
C9—C8—C14—C15	−178.0 (2)	C3—O3A—C3A—O3B	2.5 (5)
C7—C8—C14—C13	−179.37 (19)	C3—O3A—C3A—C3B	−177.4 (3)
